# Classification of Inherited Retinal Diseases Using Artificial Intelligence Models for Fundus Autofluorescence and Ultrawide Retinal Images

**DOI:** 10.1155/joph/8810684

**Published:** 2026-07-31

**Authors:** Han Trinh, Muhammad Ibrahim, Jason Charng, Zahra Tajbakhsh, Fred K. Chen, Ajmal Mian, Khyber Alam

**Affiliations:** ^1^ Department of Optometry and Vision Sciences, School of Health and Clinical Sciences, The University of Western Australia, Crawley, Western Australia, Australia, uwa.edu.au; ^2^ School of Physics, Maths and Computing, Computer Science and Software Engineering, The University of Western Australia, Nedlands, Western Australia, Australia, uwa.edu.au; ^3^ Centre for Ophthalmology and Visual Sciences (Incorporating Lions Eye Institute), The University of Western Australia, Nedlands, Western Australia, Australia, uwa.edu.au; ^4^ Ophthalmology, Department of Surgery, The University of Melbourne, East Melbourne, Victoria, Australia, unimelb.edu.au; ^5^ Royal Victorian Eye and Ear Hospital, East Melbourne, Victoria, Australia, eyeandear.org.au

## Abstract

**Background/Objectives:**

Inherited retinal diseases (IRDs) are a leading cause of blindness in working‐age adults. Although artificial intelligence (AI) shows potential for disease classification, progress is limited by small datasets, reliance on labelled data and limited integration of multiple imaging modalities. RETFound, a foundation model pretrained on over 900,000 fundus photographs, may address these limitations. While RETFound is based on the transformer architecture, convolutional neural networks (CNNs), such as ResNet and EfficientNet_B0, have also demonstrated strong performance in classifying retinal diseases. This study adapted RETFound and CNNs for analysing fundus autofluorescence (FAF) and pseudocolour ultra‐widefield (UWF) images, establishing a framework to classify IRDs.

**Subjects/Methods:**

Deidentified FAF and UWF images were obtained from controls and patients with Best disease, rod‐cone dystrophy, Stargardt disease and choroideremia. Images underwent preprocessing and were used to fine‐tune RETFound. Model performance was compared with classical machine learning algorithms (logistic regression, support vector machine, gradient boosting and random forest) and deep learning architectures (ResNet18, ResNet50, vision transformer, EfficientNet_B0 and ConvNeXt‐Tiny) pretrained on ImageNet.

**Results:**

The fine‐tuned RETFound model achieved an accuracy of 0.815, with the best performance for rod‐cone dystrophy (F1 = 0.820). RETFound outperformed classical machine learning models and a vision transformer pretrained on ImageNet. ResNet18 achieved a weighted F1 of 0.839 and demonstrated the best classification performance for Stargardt disease and choroideremia (F1 0.821 and 0.728, respectively). ResNet50 achieved a weighted F1 of 0.825 and demonstrated the best classification performance for normal retinas and Best disease (F1 0.945 and 0.616, respectively). ResNet architectures achieved the best performance overall.

**Conclusions:**

ResNet architectures and fine‐tuned RETFound both demonstrated strong accuracy in classifying IRD classes, demonstrating potential for clinical application and deployment in eye care settings as tools for IRD diagnosis, triage and management.

## 1. Introduction

Inherited retinal diseases (IRDs) are a group of heterogeneous genetic ocular conditions that cause vision impairment due to degeneration, dysfunction or abnormal development of retinal layers. IRDs affect 0.029% of the global population [1], or 5.5 million people worldwide[2], and is the leading cause of blindness in working‐age adults [3]. Over 300 genes have been implicated in IRD [4], leading to diverse pathophysiology, heterogeneous presentations and clinical challenges in managing uncertainties in disease progression.

The use of artificial intelligence (AI) has risen in popularity in recent years, particularly in health care fields. AI models infer patterns from data to make predictions on unseen cases, with model design depending on data modality and output type. For example, some AI models, such as convolutional neural networks (CNNs) [5], were designed to process image‐based data, while other models, such as transformers [6], were designed for processing text‐based data. Modern neural network‐based architectures, such as CNNs and transformers, have surpassed classical machine learning techniques such as logistic regression and support vector machines (SVMs) [7].

CNNs, such as Residual Networks (ResNet) [8] and EfficientNet [9], are commonly used in the field of eye care due to their ability to learn patterns within large image‐based datasets, which holds significant clinical value. ResNet is able to learn both fine details and broader patterns in images [8]. ResNet50, a ResNet architecture with 50 layers, classified fundus images of normal retinas, age‐related macular degeneration (AMD), and tessellated fundi with an accuracy of up to 93.81% [10] and outperformed other CNNs in diabetic retinopathy detection [11]. EfficientNet achieves high performance with lower computational cost by scaling network depth, width and resolution [12] and has been used extensively to detect diabetic retinopathy [13–17] and glaucoma [18–20] and to accurately classify various ocular conditions, such as glaucoma and cataract [21]; and retinal vein occlusion, high myopia, glaucoma, diabetic retinopathy and macular degeneration [22].

In IRD research, ResNet101 accurately classified fundus autofluorescence (FAF) images of various IRDs (including Stargardt disease, retinitis pigmentosa and Best disease) [23]. Visual Geometry Group 16 (VGG‐16) [24] has been used to distinguish between normal and retinitis pigmentosa FAF and pseudocolour ultra‐widefield (UWF) retinal images [25], and VGG‐19 was used to classify optical coherence tomography (OCT) images as normal, mild or severe Stargardt disease [26]. Automated IRD classification has the potential to support clinical diagnosis and screening, particularly given the heterogeneous nature of IRD presentations.

Although there is a growing body of literature that utilises AI in the field of IRD, there remain several methodological challenges and limitations. Supervised learning approaches ‐ that is, AI models that learn representations by finding patterns in labelled data ‐ such as CNNs, require large amounts of high‐quality data tagged with specific categories for training. This requires time and expertise to manually assign labels, and label accuracy is clinician‐dependent. Furthermore, there is limited data for IRDs due to their rarity compared to more common ophthalmic conditions such as diabetic retinopathy, AMD and glaucoma. Additionally, many studies in this area use training sets comprising only one imaging modality, such as OCT [26, 27], colour fundus photo [28, 29] or FAF [23, 30, 31] individually, which may overlook the clinical value that multimodal imaging provides.

Foundation models have emerged to help overcome the methodological limitations of scarcelabelled data and lack of multimodal imaging. RETFound [32] is a foundation model for the retina and is based on the vision transformer architecture. It was trained on a large amount of unlabelled data (904,170 colour fundus photographs and 736,442 OCT images) from normal patients and people with various ocular conditions, using various types of equipment from numerous sites worldwide. Compared to supervised AI models, RETFound demonstrates superior performance in downstream tasks, including detecting ocular conditions such as glaucoma and diabetic retinopathy, and predicting systemic diseases from fundus photos [32]. Studies have applied the concepts of foundation models and expanded on RETFound. RetiZero [33] links images with clinical terminology by integrating RETFound with a framework that enables learning from both fundus images and disease‐related textual descriptions. Compared to image‐based RETFound alone, the inclusion of text‐based information led to a 9.40% improvement in Top‐1 accuracy (a measure of how often a model’s most confident prediction is correct) for classifying 15 retinal conditions. Similarly, RetFiner combined RETFound with a text encoder to enable joint processing of OCT images and their corresponding textual descriptions [34]. This resulted in superior performance in disease classification of OCT images compared to RETFound. Furthermore, the potential of synthetic data for RETFound training has been explored [35]. Specifically, one million synthetic colour fundus photographs were generated and used in place of most real‐world data, requiring only 16.7% of the 904,170 original fundus images to achieve similar performance to RETFound. The modifications made on RETFound demonstrate its generalisability and highlight the potential of foundation models in IRD research.

Since RETFound is highly adaptable and easy to modify for downstream tasks, it has further potential to be adapted to IRDs. RETFound was trained on colour fundus photographs and OCT scans. While these imaging modalities provide valuable clinical information, in the context of IRDs, many diagnostic signs are more evident on FAF compared to colour fundus photos, such as hyper‐ and hypoautofluorescent flecks in Stargardt disease visible with FAF [36], or hypoautofluorescent patches of retinal atrophy that may only appear subtly discoloured on fundus photographs [37]. Incorporating FAF may improve RETFound’s performance and applicability to IRDs. Despite the diagnostic relevance of FAF and UWF imaging in IRDs, to date, no foundation models have been developed for these imaging modalities. This study therefore aimed to (1) develop a dataset comprising pseudocolour and FAF UWF images, titled UWA‐IRD dataset; (2) adapt RETFound for FAF and pseudocolour UWF retinal images to establish a foundation model framework for IRD classification; and (3) apply CNN architectures to IRD images, given their accuracy in ocular disease image classification.

## 2. Methods

This study was approved by the ethics committee of the University of Western Australia (2025/ET000737) and adhered to the tenets of the Declaration of Helsinki. Retrospective deidentified normal data were extracted from patients seen at the Eye Health Centre of Western Australia (EHCWA) and patients enrolled in the Western Australian Retinal Degeneration study (WARD) [38] at the Lions Eye Institute (LEI). Retrospective deidentified IRD data were extracted from WARD participants. All patients had provided written informed consent for their deidentified data to be used for research.

## 3. Image Collection for UWA‐IRD Dataset

Deidentified pseudocolour and FAF UWF images of normal retinas and retinas with IRD were extracted. For LEI patients, pseudocolour and FAF UWF images were obtained using a confocal scanning laser ophthalmoloscope (Optos California P200DTx, Optos plc, Dunfermline, Scotland) following pupil dilation. For EHCWA patients, pseudocolour and FAF UWF images were obtained using Optos, either undilated or with pupil dilation when undilated image quality was poor. Clinical diagnoses for LEI patients and EHCWA patients were provided by retinal specialist FC and experienced optometrists, respectively. Diagnoses were made based on genotype and clinical phenotype. All clinical diagnoses were reviewed and confirmed by optometrist HT.

Exclusion criteria for normal images were (1) any signs of retinal disease visible on pseudocolour/FAF UWF images and (2) poor image quality (e.g., motion or defocus blur, eyelash or eyelid artefacts obscuring more than 30% of the field of view, illumination imbalance, excessive shadowing or black ring artefact and incomplete capture). Anatomical variants, such as physiological large cupping or cilioretinal arteries, were included. IRD exclusion criteria were (1) concomitant nonretinal ocular conditions visible on pseudocolour/FAF UWF imaging (for example, advanced cataract, corneal opacities and dense and/or prolific floaters) and (2) poor image quality.

### 3.1. Genetic Analysis and Pathogenicity Assessment

Genetic testing of DNA was conducted through the Australian Inherited Retinal Disease Registry [39] and DNA Bank. Genomic DNA extracted from peripheral blood was analysed by targeted next‐generation sequencing (NGS), using a retinal dystrophy NGS SmartPanel targeting all exons and flanking intronic regions of *ABCA4*, *CHM*, *BEST1* and known genes associated with rod‐cone dystrophy (RCD). Candidate variants and mutations in parents and other affected siblings were confirmed by Sanger sequencing (Casey Eye Institute Molecular Diagnostics Laboratory, Portland, OR, USA or Molecular Vision Laboratory, Hillsboro, OR, USA). Variant nomenclature was reported in accordance with the Human Genome Variation Society [40]. Pathogenicity was assessed and interpreted according to the American College of Medical Genetics and Genomics [41] and Association for Molecular Pathology [42].

### 3.2. Image Extraction and Preprocessing

FAF and pseudocolour UWF images were downloaded and processed offline. For each patient, one high‐quality FAF and one high‐quality pseudocolour image were selected per eye, drawn from any available clinic visit. Both right and left eyes were included when suitable images were available, and data splitting was performed at the patient level to ensure that the patients in the training and test sets were mutually exclusive and to ensure no data leakage. Each patient was represented in the dataset only once per eye, per modality.

### 3.3. Black Border Removal and Retinal Region Extraction

Raw Optos UWF images were acquired at approximately 4000 × 4000 pixels, encompassing a 200‐degree field of view with black borders surrounding the retinal area. Pseudocolour images comprised red, green and blue channels, and FAF images comprised one greyscale channel. An automated border removal algorithm was applied: each image was converted to greyscale, thresholded to separate the retinal content from the dark surround and converted into a binary mask to identify the main retinal area. The retinal region and a tight bounding box with an 8‐pixel margin were extracted.

### 3.4. Optic Disc‐Centred Cropping and Spatial Downsampling

A 1024 × 1024‐pixel region was cropped from each raw image, centred on the optic nerve (manually identified). Manual identification was performed consistently by a single grader to minimise inter‐observer variability. Although automated optic nerve detection algorithms exist, manual localisation was selected to ensure accurate centring in cases with atypical anatomy or pathology (e.g., peripapillary atrophy or distorted disc margins), which can challenge automated approaches. The 1024 × 1024 pixel crop size was deliberately chosen to capture a wider retinal area around the optic disc compared to conventional tight crops (e.g., 228 × 228 pixels), thereby preserving diagnostically relevant peripheral features such as pigmentary changes in RCD and fleck patterns in Stargardt disease. The 1024 × 1024 crops were then spatially downsampled to 256 × 256 pixels, selecting every fourth pixel along both spatial dimensions (i.e., a stride‐4 subsampling). This approach was preferred over interpolation‐based resizing (e.g., bilinear or bicubic) to maintain high‐frequency structural details such as lesion boundaries that are critical for disease discrimination.

### 3.5. Multimodal Image Fusion

A channel‐level multimodal fusion strategy was employed to combine the complementary information from FAF and RG modalities into a single three‐channel input suitable for standard convolutional architectures. When only one modality was available, graceful fallback strategies were applied: for RG‐only samples, the FAF channel was replaced by a luminance‐weighted greyscale conversion of the RG image; for FAF‐only samples, the three‐channel image was formed by replicating the FAF image across all channels. To ensure consistent laterality, all left‐eye images were horizontally flipped to match right‐eye orientation prior to fusion.

### 3.6. Data Augmentation

#### 3.6.1. Spatial Augmentation: Random Shift Cropping

Each training image was slightly shifted by ± 2 pixels in both horizontal and vertical directions (implemented via reflective padding), followed by a centre crop of 252 × 252 pixels and bilinear resizing to the model input resolution of 224 × 224. This provides subtle positional variation that improves model robustness without introducing geometric distortions that could alter clinically relevant spatial relationships. Test images did not undergo any spatial augmentation.

#### 3.6.2. Photometric Augmentation

Small adjustments were made to brightness, contrast and gamma for training images in order to increase variation while maintaining the clinical relevance of images. Contrast Limited Adaptive Histogram Equalisation was applied to FAF channels to enhance local contrast and improve visibility of subtle autofluorescence patterns.

#### 3.6.3. Synthetic Data Generation via Pixel‐Space Interpolation

To address class imbalance and augment the limited training set, an offline synthetic data generation strategy inspired by synthetic minority over‐sampling technique [43] was employed. For each disease class, 20 synthetic image pairs were generated per fold by randomly selecting two real training samples from the same class and creating a blended image. This interpolation was applied independently to both RG and FAF modalities when both were available. The resulting synthetic images exhibit plausible intermediate disease appearances that expand the intra‐class variation observed during training. Synthetic images were used exclusively in the training set.

#### 3.6.4. MixUp Regularisation

During training, MixUp augmentation [44] was applied. Pairs of training images were randomly blended together in order to increase training sample size and encourage the model to reduce overconfident predictions on the small training set.

### 3.7. Model Architecture

#### 3.7.1. RETFound: Retinal Foundation Model (Proposed)

The proposed classification framework is built upon RETFound, a Vision Transformer (ViT‐Large, patch size 16, input resolution 224 × 224), that was pretrained using masked autoencoder (MAE) self‐supervised learning on a large‐scale retinal image corpus of 904,170 colour fundus photographs and 736,442 OCT images. RETFound’s original output layer was replaced with a simpler layer that classifies images into five categories, with a dropout layer added to reduce overfitting. The entire model was then fine‐tuned on the present IRD dataset.

### 3.8. Training Procedure

All models were trained using the AdamW optimiser [45] (learning rate = 2 × 10^−4^, weight decay = 0.05) with cosine annealing learning rate scheduling over a maximum of 60 epochs. Mixed‐precision training (FP16) was enabled via PyTorch’s automatic mixed precision for computational efficiency. Gradient clipping with a maximum norm of 1.0 was applied. Class‐weighted cross‐entropy loss [46] was used, where the weight for each class was computed as the ratio of the maximum class count to that class’s count in the training set, addressing the significant class imbalance (normal: 181 eyes vs. Best disease: 32 eyes). A class‐balanced weighted random sampler was employed to ensure equal expected class representation per epoch. Early stopping with a patience of 12 epochs was applied based on the validation macro F1‐score, and the best checkpoint was selected for final test evaluation.

The weighted random sampler was used to balance how samples were distributed across batches to address dataset imbalance. The batch size defines how many samples are used in each training iteration, while epochs indicate how many times the model passes through the entire training dataset. The loss function measures the difference between the model’s predictions and the ground truth, and the optimiser adjusts the model’s parameters to minimise this loss and improve prediction accuracy.

An ablation study was conducted to systematically evaluate and validate the contribution of each component (inclusion of augmented images during training, inclusion of synthetic images during training, and contribution of individual imaging modalities) to model performance.

### 3.9. Comparison Models

To contextualise the performance of the proposed RETFound‐based framework, nine baseline models spanning classical machine learning and deep learning architectures were evaluated under identical data splits and preprocessing conditions. Model architectures were selected because they are widely used for retinal image classification, represent both convolutional and transformer‐based approaches and are the dominant architectures in ophthalmic image analysis. Hyperparameters, including batch size, number of epochs and optimiser choice, were selected based on commonly used settings in prior deep learning studies in ophthalmic imaging and were chosen to ensure stable training within available computational resources.

#### 3.9.1. Deep Learning Baselines

Five convolutional and transformer architectures were trained from ImageNet‐pretrained initialisations: ResNet‐18, ResNet‐50, EfficientNet‐B0, ViT‐Base/16 and ConvNeXt‐Tiny. Each model was fine‐tuned for a maximum of 40 epochs using AdamW (learning rate = 2 × 10^−4^, weight decay = 0.05) with cosine annealing, class‐weighted cross‐entropy loss and early stopping (patience = 10 epochs) on validation macro F1.

#### 3.9.2. Classical Machine Learning Baselines

Four classical machine learning algorithms were evaluated: logistic regression, balanced class weights, SVM, random forest and gradient boosting. For these models, each preprocessed 224 × 224 × 3 image was flattened to a 150,528‐dimensional feature vector, standardised with *z*‐score normalisation, and reduced to 200 principal components via principal component analysis. Training and validation sets were combined for classical models, as hyperparameter selection was not performed.

### 3.10. Evaluation Protocol

#### 3.10.1. Patient‐Wise Stratified 5‐Fold Cross‐Validation

Patient‐wise stratified 5‐fold cross‐validation was employed to ensure that images from the same patient were not used in both training and testing, which would otherwise inflate performance estimates. Patients were split into five groups using stratified K‐fold splitting on patient‐level disease labels. Within each fold, the four nontest partitions were further split into training (87.5%) and validation (12.5%) subsets using an inner stratified split, which is a technique used to partition data into subsets while preserving the original proportion of each class label in each subset. This yielded an approximate 70/10/20 train/validation/test % distribution per fold. The splits were kept the same across all models to ensure fair comparison.

#### 3.10.2. Trained Model Inference

For disease classification, the optic nerve centre was manually identified on test images by a clinician experienced in retinal image interpretation and used to centre and crop test images to a 224 × 224 size (three channels, using methods described in Section 3.4). Cropped images were put through the various AI models to output predicted disease classification.

**FIGURE 1:**
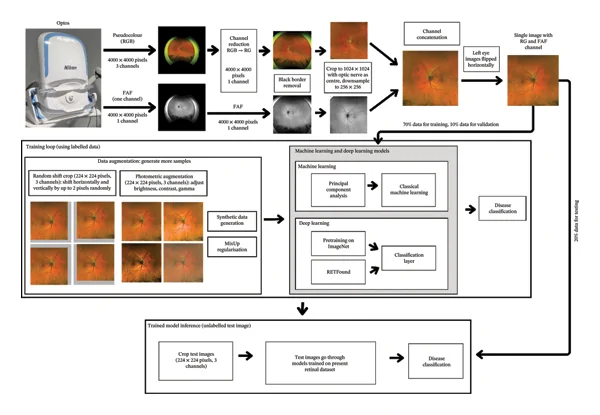
Workflow and experiment setup. Pseudocolour and fundus autofluorescence images were extracted from Optos software, and black borders were removed. Images were cropped to align the centre of the optic nerve with the centre of the image and downsampled. Channels were concatenated and images were flipped horizontally when appropriate. To increase sample size, data augmentation (including random shift cropping, photometric augmentation, synthetic data generation and MixUp regularisation) was performed. Processed images were used to train various classical machine learning and deep learning models. Test images were cropped and analysed by machine learning and deep learning models, and disease classification was performed.

Figure 1 illustrates the steps of the study workflow.

### 3.11. Model Performance Metrics

Four complementary metrics were computed on each test fold: overall accuracy, macro‐averaged F1‐score (unweighted across classes), weighted F1‐score (weighted by class support), and macro‐averaged area under the receiver operating characteristic curve (AUC, one‐vs.‐rest). Per‐class precision, recall and F1‐score were also reported. All metrics were aggregated across the five folds, and mean ± standard deviation values are reported.

Classification performance was assessed using class‐specific mean F1 scores, weighted F1 scores and AUC. For comparisons across models, AUC and macro F1 score were considered, which provides a truer representation of performance on minority classes compared to weighted F1. For clinical comparisons, precision and F1 score were considered.

## 4. Results

### 4.1. Dataset

The normal retina group comprised 20 FAF and 181 pseudocolour images from 181 eyes of 101 patients (52 female, 49 male) with a mean age of 33.05 ± 14.01 years (median age 27 years, range 18–74 years) and a mean logMAR visual acuity of −0.06 ± 0.06 at image acquisition. Images of insufficient quality and images from visits with incomplete data (for example, no record of visual acuity) were discarded.

The diseased retina group comprised four classes—Best disease, choroideremia, RCD and Stargardt disease. Patient and image characteristics are summarised in Table 1. The Best disease class comprised 32 FAF and 32 pseudocolour UWF images from 32 eyes of 16 patients (10 female, 6 male) with a mean age of 42.5 ± 12.8 years (median age 47 years, range 26–71 years) and mean logMAR visual acuity of 0.30 ± 0.33 at image acquisition. The choroideremia class comprised 38 FAF and 38 pseudocolour UWF images from 38 eyes of 19 patients (12 female, 7 male) with a mean age of 49.16 ± 20.61 years (median age 48 years, range 19–86 years) and a mean logMAR visual acuity of 0.25 ± 0.47. The RCD class comprised 81 FAF and 79 pseudocolour UWF images from 82 eyes of 42 patients (26 female, 16 male) with a mean age of 45.29 ± 15.17 years (median age 48 years, range 21–67 years) and mean logMAR visual acuity of 0.37 ± 0.46 at image acquisition. The Stargardt disease class comprised 56 FAF and 56 pseudocolour UWF images from 56 eyes of 28 patients (16 female, 12 male) with a mean age of 53.86 ± 20.45 years (median age 53 years, range 16–90 years) and a mean logMAR visual acuity of 1.11 ± 0.70 at image acquisition.

**TABLE 1:** Summary of patient characteristics and raw imaging data used in this study.

Class	# Patients	Genotype [number of patients]	# Eyes	Mean patient age (years)	# UWF pseudocolour images	# UWF FAF images	Mean visual acuity (logMAR)
Normal	101	N/A	181	33.05 ± 14.01	181	20	−0.06 ± 0.07

Best disease	16	*BEST1* [16]	32	42.5 ± 12.8	32	32	0.30 ± 0.33

Choroideremia	19	*CHM* [19]	38	49.16 ± 20.61	38	38	0.25 ± 0.47

Rod‐cone dystrophy	42	*RP1* [5] *USH2A* [8] *RHO* [5] *PRPF31* [6] *EYS* [2] *RGR* [1] *CNGA1* [1] *PRPH2* [2] *RP9* [1] *CLN3* [1] *HGSNAT* [1] *HK1* [1] *IMPDH1* [1] *RPGR* [1] *CRB1* [1] *SPATA7* [1] *PDE6B* [1] *COL4A5* [1] *VPS13B + USH2A* [1] *LRP4 + BFSP1 + SETX* [1]	82	45.29 ± 15.17	81	79	0.37 ± 0.46

Stargardt disease	28	*ABCA4* [28]	56	53.86 ± 20.45	56	56	1.11 ± 0.70

*Note:* FAF, fundus autofluorescence; UWF, ultra‐widefield.

Of the 389 eye‐level samples, 223 (57.3%) had both FAF and RG modalities, 164 (42.2%) had RG only and 2 (0.5%) had FAF only. Left and right eyes were approximately balanced (194 left, 195 right).

**FIGURE 2:**
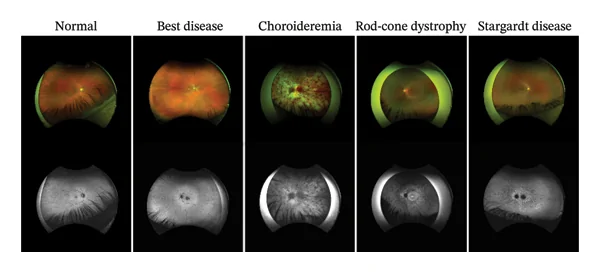
Samples from the UWA‐IRD dataset. The top row contains representative pseudocolour ultra‐widefield retinal images for each class; the bottom row contains representative fundus autofluorescence images for each class. Images were taken from both right and left eyes.

Samples of the UWA‐IRD dataset are represented in Figure 2.

### 4.2. Overall Model Comparison

Table 2 presents the comparative performance of all 10 evaluated models across the six metrics, aggregated over the five cross‐validation folds. The proposed RETFound‐based model achieved an accuracy of 0.815 ± 0.049, a macro F1‐score of 0.730 ± 0.065 and an AUC of 0.945 ± 0.012. Among the comparison models, ResNet‐18 achieved the highest accuracy (0.833 ± 0.039) and macro F1 (0.765 ± 0.047), followed by ResNet‐50 (accuracy 0.823 ± 0.040, macro F1 0.739 ± 0.055) and EfficientNet‐B0 (accuracy 0.807 ± 0.013, macro F1 0.714 ± 0.031). All deep learning models outperformed the classical machine learning baselines, with random forest displaying the lowest performance (accuracy 0.532 ± 0.033, macro F1 0.235 ± 0.036).

**TABLE 2:** Comparative performance of all models (5‐fold cross‐validation, mean ± standard deviation).

Model	Accuracy	F1 (macro)	F1 (weighted)	AUC (macro)
RETFound (fine‐tuned on the UWA‐IRD dataset)	0.815 ± 0.049	0.730 ± 0.065	0.818 ± 0.045	**0.912 ± 0.012**
ResNet‐18	**0.833 ± 0.039**	**0.765 ± 0.047**	**0.839 ± 0.034**	**0.941 ± 0.009**
ResNet‐50	0.823 ± 0.040	0.739 ± 0.055	0.825 ± 0.040	0.933 ± 0.022
EfficientNet‐B0	0.807 ± 0.013	0.714 ± 0.031	0.808 ± 0.013	**0.954 ± 0.014**
ConvNeXt‐Tiny	0.774 ± 0.038	0.685 ± 0.044	0.784 ± 0.035	0.911 ± 0.034
ViT‐B/16	0.717 ± 0.045	0.599 ± 0.054	0.722 ± 0.044	0.882 ± 0.024
Logistic regression	0.759 ± 0.068	0.626 ± 0.097	0.760 ± 0.070	0.922 ± 0.041
SVM	0.720 ± 0.034	0.586 ± 0.039	0.722 ± 0.035	0.890 ± 0.023
Gradient boosting	0.663 ± 0.046	0.441 ± 0.062	0.608 ± 0.053	0.853 ± 0.017
Random forest	0.532 ± 0.033	0.235 ± 0.036	0.411 ± 0.046	0.878 ± 0.027

*Note:* Best performance is highlighted in bold.

### 4.3. Discriminative Performance

**FIGURE 3:**
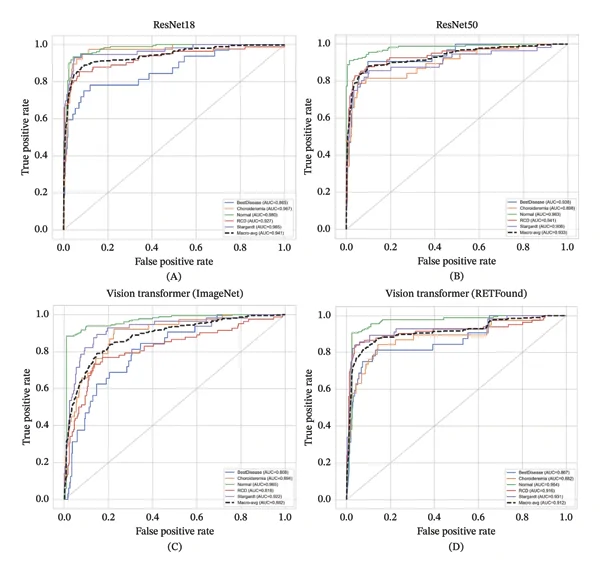
Receiver operator curves for disease classification performance of various deep learning models: (A) ResNet18; (B) ResNet50; (C) a vision transformer pretrained on ImageNet; (D) the fine‐tuned RETFound model.

The proposed RETFound model achieved a macro‐averaged AUC of 0.912 ± 0.012. ResNet‐18 achieved an AUC of 0.941 ± 0.009, and ResNet50 achieved 0.933 ± 0.022 (Figure 3). EfficientNet_B0 achieved an AUC of 0.954 ± 0.014, while ConvNeXt‐Tiny and ViT‐B/16 showed lower discriminative performance, with AUCs of 0.911 ± 0.034 and 0.891 ± 0.024, respectively. ROC curves for the top four performing models are presented in Figure 3.

Per‐class analysis revealed that classification performance was highest for the normal class (F1 score 0.945 with ResNet‐50), followed by RCD and Stargardt disease (F1 scores of 0.820 and 0.821 with RETFound and ResNet‐18, respectively), choroideremia (F1 score 0.728 with ResNet‐18) and Best disease (F1 score 0.616 with ResNet‐50) (Table 3).

**TABLE 3:** Performance comparison of the current IRD classification framework across classical machine learning and convolutional neural network (CNN) architectures on the UWA‐IRD dataset.

Method	Normal	Best disease	Choroideremia	Rod‐cone dystrophy	Stargardt disease
EfficientNet_B0	0.961/0.941	**0.590**/0.525	0.515/0.611	0.767/0.766	0.793/0.727
ResNet‐18	0.971/0.928	0.461/0.535	**0.729/0.728**	0.856/0.813	**0.824/0.821**
ResNet‐50	**0.989/0.945**	0.519/**0.616**	0.677/0.628	0.824/0.806	0.701/0.703
ViT‐B/16	0.982/0.930	0.306/0.322	0.491/0.523	0.666/0.546	0.596/0.672
ConvNeXt‐Tiny	0.964/0.925	0.360/0.414	0.604/0.638	0.788/0.715	0.730/0.730
RETFound	0.950/0.930	0.520/0.550	0.670/0.610	**0.880/0.820**	0.690/0.760

*Note:* Each cell reports the precision/F1 score. The best result per class is bolded. Classical machine learning architectures (logistic regression, support vector machine, random forest and gradient boosting) utilised principal component analysis. CNNs (EfficientNet_B0, ResNets and ConvNeXt‐Tiny) and vision transformers were pretrained on ImageNet. RETFound was pretrained on the RETFound retinal dataset.

## 5. Discussion

The present study evaluated the performance of a fine‐tuned retinal foundation model, RETFound, as well as various CNN architectures pretrained on ImageNet, for IRD and retinal disease classification using pseudocolour and FAF UWF images. RETFound, fine‐tuned on FAF and UWF images, achieved strong performance, particularly in classifying RCD (*F*1 = 0.820). The fine‐tuned RETFound model consistently outperformed classical machine learning approaches such as logistic regression and SVM across all classes, demonstrating the advantage of deep learning approaches that automatically extract relevant image features rather than relying on hand‐crafted, labelled features. ResNet architectures also performed well in classifying normal retinas, choroideremia and Stargardt disease (*F*1 = 0.945, 0.728 and 0.821, respectively), demonstrating that these well‐established CNNs can be effectively applied to smaller IRD datasets and remain clinically useful.

A key finding is that using a model trained specifically on retinal images achieved better classification performance compared to pretraining on general image datasets such as ImageNet. The fine‐tuned RETFound model, pretrained on retinal data, outperformed a vision transformer pretrained on ImageNet, likely due to the limited training set size. This is consistent with previous literature. For example, a recent study [47] demonstrated that RETFound outperformed models (including a vision transformer and VGG16) pretrained on ImageNet when identifying cup‐disc ratio from fundus photos and predicting RNFL thickness from OCT—tasks outside its original training. This suggests that RETFound’s prior knowledge of the retina allows the model to capture relevant features more accurately than models pretrained on general images. These findings also demonstrate the generalisability of RETFound even for novel tasks [47] and untrained imaging modalities, as in the present study.

Concatenation, or fusion, of multiple imaging modalities has been utilised in the field of medical imaging to enhance feature richness [48]. By concatenating features, algorithms are able to learn from multiple types of information simultaneously. In eye care, information from fundus images and OCT has been concatenated to train a framework with 98.9% accuracy in classifying normal retinas and images of AMD, diabetic retinopathy and macular hole [49] and to classify multiple retinal and systemic disease classes, demonstrating improved performance with concatenation (AUC 0.855) compared to single‐modality methods (AUC 0.729) [50]. One study concatenated features from FAF and pseudocolour UWF retinal images to predict visual function (visual acuity, central visual field sensitivity and mean deviation) in eyes with retinitis pigmentosa [51]. Although the model in that study did not demonstrate robust accuracy compared to ground truths, with regression coefficients of 0.684, 0.697 and 0.309 for predicting mean deviation, central visual field sensitivity and visual acuity, respectively, it demonstrates the potential of feature concatenation in exploring multimodal disease markers and structure–function relationships.

Another observation from this study is that ResNet, pretrained on ImageNet, outperformed the fine‐tuned RETFound model for certain disease classes. This difference likely reflects both architectural characteristics and dataset demands. Transformers are designed to capture global relationships across entire images [32] and typically require larger datasets to maximise performance due to more complex architectures compared to deep learning models. With small datasets, as in the case of the present study, the limited number of training examples may not provide sufficient variability for transformers to learn robust global representations, increasing the risk of overfitting. CNNs such as ResNet are highly effective at capturing local, fine image details [52], such as subtle pigment clumping or small hyperautofluorescent spots, which are often critical for classifying certain IRDs. The relative performance of RETFound and ResNet depends on a combination of disease‐specific feature requirements, dataset size and imaging characteristics. Rather than demonstrating superior performance across all classes, RETFound performs best when global retinal context is informative, while CNNs may outperform in tasks reliant on precise local detail. In this study, images were cropped to the optic nerve, macula and mid‐peripheral region, which may have favoured CNN‐based feature detection in some cases. CNN‐based hybrid architectures have been explored in the literature to classify various macular conditions on OCT using a CNN combined with random forest [53] and to detect retinal conditions on large OCT datasets using squeeze‐and‐excitation blocks combined with CNNs [54]. Hybrid architectures may be a way to combine CNNs’ local precision with RETFound’s global analysis to improve performance across all disease classes. Prior studies have demonstrated that deep learning models can achieve sensitivity and specificity comparable to, or in some cases exceeding, expert clinicians in diabetic retinopathy screening [55, 56] and detection of retinal detachment [57] and fluid associated with macular degeneration [58]. In this context, the performance metrics reported in the present study fall within ranges that have been considered clinically meaningful in similar applications.

Despite strong overall results, classification performance for Best disease was notably lower, likely due to its small sample size (16 patients) and phenotypic variability. Best disease can present with diverse clinical manifestations, and, when associated with subfoveal vitelliform lesions, it can masquerade as central serous chorioretinopathy [59]. Recessive bestrophinopathy has multifocal vitelliform lesions, subretinal neovascularisation and extensive intraretinal fluid which bears no resemblance to the phenotype seen in dominant vitelliform macular dystrophy or vitreoretinochoroidopathy. This phenotypic overlap and variability may have limited the model’s ability to learn consistent and class‐specific features. En face imaging modalities such as FAF and UWF imaging may not capture the most discriminative disease characteristics, as the key features in Best disease are predominantly depth‐dependent (for example, vitelliform material, intraretinal fluid, subretinal fluid and outer retinal/RPE alterations), which can be visualised on OCT.

Due to the rare nature of IRDs, sample size is inherently limited. To partially overcome this, the effective training set was increased by including imaging from both eyes, applying multiple data augmentation strategies, using weighted sampling for class imbalance, and incorporating synthetic image generation for under‐represented classes. However, these strategies may introduce limitations such as potential inter‐eye correlation and data leakage from eye‐level dataset splitting, residual class imbalance, potential reliance on synthetic image features, and constrained representation of full phenotypic and genotypic heterogeneity within broad diagnostic IRD categories. The limited dataset size also restricted statistical comparison between models.

Future studies should incorporate larger multicentre cohorts with patient‐level dataset splitting to minimise data leakage and better capture disease heterogeneity. Additional analyses should explore alternative methods for class imbalance, such as class‐weighted loss functions [60] or ensemble approaches [61]; perform formal statistical comparisons between models; and assess external validation across diverse institutions.

Clinically, this study highlights the potential for RETFound and ResNet to be translated into eye care settings as supportive diagnostic tools. In primary eye care settings, such models could be used as screening support tools to flag patients with retinal images suspicious for IRDs, thereby assisting clinicians in detecting and referring relevant cases and in remote screening and triage. In secondary and tertiary clinics, AI models could complement clinicians by triaging large image volumes and prioritising cases, helping to streamline clinical review workflows. In specialist clinics, the models could be used as a second‐opinion decision support tool, provide assistance to clinicians interpreting multiple genetic variants of uncertain significance, and be used for ongoing patient care and management.

Importantly, both RETFound and ResNet achieved reliable performance despite limited data, underscoring their suitability for rare diseases. Future work should expand dataset size and diversity for improved generalisability and explore hybrid CNN‐transformer architectures. Explainable AI techniques such as gradient‐based saliency maps [62] could help to identify retinal regions contributing most strongly to model predictions. Such approaches may provide insight into whether the models rely on clinically meaningful features and could increase clinician trust in AI‐assisted decision support tools. Overall, this research presents a clinically relevant and computationally adaptable framework for advancing AI‐assisted diagnosis and monitoring of IRDs.

## 6. Conclusion

This study demonstrates that a fine‐tuned RETFound model and CNN architectures such as ResNet can achieve strong performance in classifying IRDs using pseudocolour and FAF UWF images. It highlights the generalisability of retinal foundation and convolutional frameworks to imaging modalities not previously used in foundation models. Importantly, these architectures achieved reliable performance even with limited data, supporting their suitability for rare diseases. As a proof‐of‐concept, these findings demonstrate the potential of foundation and convolutional models to support automated diagnosis and monitoring of rare retinal diseases. However, these models are not yet ready for clinical deployment, and further validation using larger multicentre datasets and prospective clinical evaluation will be necessary before implementation in real‐world settings.

## Author Contributions

Han Trinh was involved in conceptualisation, methodology, data curation and writing (original draft). Muhammad Ibrahim was involved in conceptualisation, methodology, software and writing (review and editing). Jason Charng was involved in conceptualisation, writing (review and editing) and supervision. Zahra Tajbakhsh was involved in writing (review and editing) and supervision. Fred K. Chen was involved in resources and writing (review and editing). Ajmal Mian was involved in methodology, software, writing (review and editing) and supervision. Khyber Alam was involved in conceptualisation, methodology, writing (review and editing) and supervision.

## Funding

Open access publishing was facilitated by The University of Western Australia, as part of the Wiley–The University of Western Australia agreement via the Council of Australasian University Librarians.

## Conflicts of Interest

The authors declare no conflicts of interest.

## Supporting Information

Additional supporting information can be found online in the Supporting Information section.

## Supporting information


**Supporting Information** Table S1. Ablation study of RETFound components: effect of imaging modality, augmentation, synthetic data and MixUp on classification performance. Each cell reports the metric value obtained by the corresponding RETFound configuration; the signed change (Δ) relative to the full baseline (top row, shaded) is shown in parentheses beneath in green when the ablation improves and red when it degrades performance. The first block varies the input imaging modality (FAF‐only and RG‐only); the second block (shaded) removes one training component at a time (augmentation, synthetic data or MixUp). The best value in each metric column is shown in bold. FAF = fundus autofluorescence; RG = red–green channels; Aug = data augmentation; Syn = synthetic data; *κ* = Cohen’s kappa; MCC = Matthews correlation coefficient; AUC Macro is the macro‐averaged one‐vs.‐rest ROC AUC.

## Data Availability

The data that support the findings of this study are available on request from the corresponding author. The data are not publicly available due to privacy or ethical restrictions.

## References

[1] Hanany M. , Shalom S. , Ben-Yosef T. , and Sharon D. , Comparison of Worldwide Disease Prevalence and Genetic Prevalence of Inherited Retinal Diseases and Variant Interpretation Considerations, Cold Spring Harbor Perspectives in Medicine. (2024) 14, no. 2, 10.1101/cshperspect.a041277. PMC10835612 37460155

[2] Hanany M. , Rivolta C. , and Sharon D. , Worldwide Carrier Frequency and Genetic Prevalence of Autosomal Recessive Inherited Retinal Diseases, Proceedings of the National Academy of Sciences. (2020) 117, no. 5, 2710–2716, 10.1073/pnas.1913179117. PMC7007541 31964843

[3] Heath Jeffery R. C. , Mukhtar S. A. , McAllister I. L. , Morgan W. H. , Mackey D. A. , and Chen F. K. , Inherited Retinal Diseases Are the Most Common Cause of Blindness in the Working-Age Population in Australia, Ophthalmic Genetics. (2021) 42, no. 4, 431–439, 10.1080/13816810.2021.1913610. 33939573 PMC8315212

[4] Daiger S. , Greenberg J. , Christoffels A. , and Hide W. , Retnet, the Retinal Information Network, https://RetNet.org/.

[5] Luo W. , Li Y. , Urtasun R. , and Zemel R. , Understanding the Effective Receptive Field in Deep Convolutional Neural Networks, Advances in Neural Information Processing Systems. (2016) 29.

[6] Vaswani A. , Shazeer N. , Parmar N. et al., Attention Is all You Need, Advances in Neural Information Processing Systems. (2017) 30.

[7] Hassan S. U. , Ahamed J. , and Ahmad K. , Analytics of Machine Learning-Based Algorithms for Text Classification, Sustainable Operations and Computers. (2022) 3, 238–248, 10.1016/j.susoc.2022.03.001.

[8] Targ S. , Almeida D. , and Lyman K. , Resnet in Resnet: Generalizing Residual Architectures, arXiv preprint arXiv:160308029. 2016.

[9] Koonce B. E.N. , Convolutional Neural Networks With Swift for Tensorflow: Image Recognition and Dataset Categorization, 2021, Springer, 109–123.

[10] Pan Y. , Liu J. , Cai Y. et al., Fundus Image Classification Using Inception V3 and ResNet-50 for the Early Diagnostics of Fundus Diseases, Frontiers in Physiology. (2023) 14, 10.3389/fphys.2023.1126780. PMC9975334 36875027

[11] Lin C.-L. and Wu K.-C. , Development of Revised ResNet-50 for Diabetic Retinopathy Detection, BMC Bioinformatics. (2023) 24, no. 1, 10.1186/s12859-023-05293-1. PMC10114328 37076790

[12] Tan M. and Le Q. , Efficientnet: Rethinking Model Scaling for Convolutional Neural Networks, *International Conference on Machine Learning*, 2019, PMLR.

[13] Arora L. , Singh S. K. , Kumar S. et al., Ensemble Deep Learning and EfficientNet for Accurate Diagnosis of Diabetic Retinopathy, Scientific Reports. (2024) 14, no. 1, 10.1038/s41598-024-81132-4. PMC11655640 39695310

[14] Maswood M. M. S. , Hussain T. , Khan M. B. , Islam M. T. , and Alharbi A. G. , Cnn Based Detection of the Severity of Diabetic Retinopathy From the Fundus Photography Using efficientnet-b5, *2020 11th IEEE Annual Information Technology, Electronics and Mobile Communication Conference (IEMCON)*, 2020, IEEE.

[15] Praneeth D. , Kumar N. S. , and Nagaraju V. , Enhanced Detection and Segmentation of Retinal Exudates in Diabetic Retinopathy Using a Feature Pyramid Network With EfficientNet-B0 Encoder, Indian Journal of Science and Technology. (2024) 17, no. 32, 3377–3387, 10.17485/ijst/v17i32.1997.

[16] Anitha J. and Sangapu S. C. , Diabetic Retinopathy Detection Using EfficientNet-Based Framework With Segmentation, *2024 3rd International Conference for Advancement in Technology (ICONAT)*, 2024, IEEE.

[17] Vijayan M. and Venkatakrishnan S. , A Regression-Based Approach to Diabetic Retinopathy Diagnosis Using EfficientNet, Diagnostics. (2023) 13, no. 4. 10.3390/diagnostics13040774 PMC9955015 36832262

[18] Toptaş B. and Hanbay D. , The Separation of Glaucoma and Non-Glaucoma Fundus Images Using EfficientNet-B0, Bitlis Eren Üniversitesi Fen Bilimleri Dergisi. (2022) 11, no. 4, 1084–1092, 10.17798/bitlisfen.1174512.

[19] Gupta N. , Garg H. , and Agarwal R. , A Robust Framework for Glaucoma Detection Using CLAHE and EfficientNet, The Visual Computer. (2022) 38, no. 7, 2315–2328, 10.1007/s00371-021-02114-5.

[20] Alkhaldi N. A. and Alabdulathim R. E. , Optimizing Glaucoma Diagnosis With Deep Learning-Based Segmentation and Classification of Retinal Images, Applied Sciences. (2024) 14, no. 17, 10.3390/app14177795.

[21] Rakib A. A. , Billah M. M. , Ahamed A. S. , Imamul H. M. , and Masum M. S. A. , EfficientNet-Based Model for Automated Classification of Retinal Diseases Using Fundus Images, European Journal of Computer Science and Information Technology. (2024) 12, no. 8, 48–61, 10.37745/ejcsit.2013/vol12n84861.

[22] Zhu S. , Lu B. , Wang C. et al., Screening of Common Retinal Diseases Using Six-Category Models Based on EfficientNet, Frontiers of Medicine. (2022) 9, 10.3389/fmed.2022.808402. PMC8904395 35280876

[23] Miere A. , Le Meur T. , Bitton K. et al., Deep Learning-Based Classification of Inherited Retinal Diseases Using Fundus Autofluorescence, Journal of Clinical Medicine. (2020) 9, no. 10, 10.3390/jcm9103303. PMC7602508 33066661

[24] Simonyan K. and Zisserman A. , Very Deep Convolutional Networks for Large-Scale Image Recognition, 2014, arXiv preprint arXiv:14091556.

[25] Masumoto H. , Tabuchi H. , Nakakura S. et al., Accuracy of a Deep Convolutional Neural Network in Detection of Retinitis Pigmentosa on Ultrawide-Field Images, PeerJ. (2019) 7, 10.7717/peerj.6900. PMC6510218 31119087

[26] Shah M. , Roomans Ledo A. , and Rittscher J. , Automated Classification of Normal and Stargardt Disease Optical Coherence Tomography Images Using Deep Learning, Acta Ophthalmologica. (2020) 98, no. 6, e715–e721, 10.1111/aos.14353. 31981283

[27] Eckardt F. , Mittas R. , Horlava N. et al., Deep Learning-Based Retinal Layer Segmentation in Optical Coherence Tomography Scans of Patients With Inherited Retinal Diseases, Klinische Monatsblätter für Augenheilkunde. (2024) . 10.1055/a-2227-3742 PMC12445938 38086412

[28] Chen T. C. , Lim W. S. , Wang V. Y. et al., Artificial Intelligence-Assisted Early Detection of Retinitis Pigmentosa-The Most Common Inherited Retinal Degeneration, Journal of Digital Imaging. (2021) 34, no. 4, 948–958, 10.1007/s10278-021-00479-6. 34244880 PMC8455770

[29] Brancati N. , Frucci M. , Gragnaniello D. et al., Learning-Based Approach to Segment Pigment Signs in Fundus Images for Retinitis Pigmentosa Analysis, Neurocomputing. (2018) 308, 159–171, 10.1016/j.neucom.2018.04.065.

[30] Charng J. , Xiao D. , Mehdizadeh M. et al., Deep Learning Segmentation of Hyperautofluorescent Fleck Lesions in Stargardt disease, Scientific Reports. (2020) 10, no. 1, 10.1038/s41598-020-73339-y. PMC7536408 33020556

[31] Charng J. , Escalona I. A. V. , Turpin A. et al., Nonlinear Reduction in Hyperautofluorescent Ring Area in Retinitis Pigmentosa, Ophthalmology Retina. (2024) 8, no. 3, 298–306, 10.1016/j.oret.2023.09.015. 37743021

[32] Zhou Y. , Chia M. A. , Wagner S. K. et al., A Foundation Model for Generalizable Disease Detection From Retinal Images, Nature. (2023) 622, no. 7981, 156–163, 10.1038/s41586-023-06555-x. 37704728 PMC10550819

[33] Wang M. , Lin T. , Yu K. et al., Common and Rare Fundus Diseases Identification Using Vision-Language Foundation Model With Knowledge of Over 400 Diseases, arXiv e-prints. 2024:arXiv: 2406.09317.

[34] Fecso R. , Morano J. , Schmidt-Erfurth U. , and Bogunović H. , RetFiner: A Vision-Language Refinement Scheme for Retinal Foundation Models, 2025, arXiv preprint arXiv:250622149.

[35] Sun Y. , Tan W. , Gu Z. et al., A Data-Efficient Strategy for Building High-Performing Medical Foundation Models, Nature Biomedical Engineering. (2025) 9, no. 4, 1–13, 10.1038/s41551-025-01365-0. 40044818

[36] Solberg Y. , Dysli C. , Escher P. , Berger L. , Wolf S. , and Zinkernagel M. S. , Retinal Flecks in Stargardt Disease Reveal Characteristic Fluorescence Lifetime Transition Over Time, Retina. (2019) 39, no. 5, 879–888, 10.1097/iae.0000000000002519. 30985551 PMC6510322

[37] Yung M. , Klufas M. A. , and Sarraf D. , Clinical Applications of Fundus Autofluorescence in Retinal Disease, International Journal of Retina and Vitreous. (2016) 2, no. 1, 10.1186/s40942-016-0035-x. PMC5088473 27847630

[38] Australian New Zealand Clinical Trials Registry , ACTRN12618000738224. The Western Australia Retinal Degeneration Study: An Natural History Observational Cohort Study of Retinal Degenerations and in Vitro Retinal Disease Modelling Using Patient Derived Stem Cells, 2018, NHMRC Clinical Trials Centre UoSA, Sydney (NSW), https://www.anzctr.org.au/Trial/Registration/TrialReview.aspx?id=374982.

[39] DeRoach J. , McLaren T. , Thompson J. , Hoffmann L. , Campbell I. , and Lamey T. , The Australian Inherited Retinal Disease Registry and DNA Bank, Clinical and Experimental Ophthalmology. (2017) Wiley, Hoboken, NJ, USA.

[40] Den Dunnen J. T. , Dalgleish R. , Maglott D. R. et al., HGVS Recommendations for the Description of Sequence Variants: 2016 Update, Human Mutation. (2016) 37, no. 6, 564–569, 10.1002/humu.22981. 26931183

[41] Richards S. , Aziz N. , Bale S. et al., Standards and Guidelines for the Interpretation of Sequence Variants: A Joint Consensus Recommendation of the American College of Medical Genetics and Genomics and the Association for Molecular Pathology, Genetics in Medicine. (2015) 17, no. 5, 405–423, 10.1038/gim.2015.30. 25741868 PMC4544753

[42] Brnich S. E. , Abou Tayoun A. N. , Couch F. J. et al., Recommendations for Application of the Functional Evidence PS3/BS3 Criterion Using the ACMG/AMP Sequence Variant Interpretation Framework, Genome Medicine. (2019) 12, no. 1, 10.1186/s13073-019-0690-2. PMC6938631 31892348

[43] Chawla N. V. , Bowyer K. W. , Hall L. O. , and Kegelmeyer W. P. , SMOTE: Synthetic Minority Over-sampling Technique, Journal of Artificial Intelligence Research. (2002) 16, 321–357, 10.1613/jair.953.

[44] Jin X. , Zhu H. , Li S. et al., A Survey on Mixup Augmentations and Beyond, 2024, arXiv preprint arXiv:240905202.

[45] Loshchilov I. and Hutter F. , Fixing Weight Decay Regularization in Adam, 2017, 5, no. 5, arXiv preprint arXiv:171105101.

[46] Mao A. , Mohri M. , and Zhong Y. , Cross-Entropy Loss Functions: Theoretical Analysis and Applications, *International Conference on Machine Learning*, 2023, PMLR.

[47] Chen M. S. , Ravindranath R. , Chang R. , Zhou Y. , Keane P. A. , and Wang S. Y. , Independent Evaluation of RETFound Foundation Model’s Performance on Optic Nerve Analysis Using Fundus Photography, Ophthalmology Science. (2025) 5, no. 3, 10.1016/j.xops.2025.100720. PMC11950761 40161459

[48] Li Y. , Daho M. E. H. , Conze P.-H. et al., A Review of Deep Learning-Based Information Fusion Techniques for Multimodal Medical Image Classification, Computers in Biology and Medicine. (2024) 177, 10.1016/j.compbiomed.2024.108635. 38796881

[49] Wang D. , Cai Y. , Qiao W. , and Liu M. , Multimodal Deep Learning for Retinal Disease Diagnosis, International Symposium on Neural Networks. (2025) Springer.

[50] He X. , Deng Y. , Fang L. , and Peng Q. , Multi-Modal Retinal Image Classification With Modality-Specific Attention Network, IEEE Transactions on Medical Imaging. (2021) 40, no. 6, 1591–1602, 10.1109/tmi.2021.3059956. 33625978

[51] Nagasato D. , Sogawa T. , Tanabe M. et al., Estimation of Visual Function Using Deep Learning from Ultra-Widefield Fundus Images of Eyes With Retinitis Pigmentosa, JAMA Ophthalmology. (2023) 141, no. 4, 305–313, 10.1001/jamaophthalmol.2022.6393. 36821134 PMC9951103

[52] He K. , Zhang X. , Ren S. , and Sun J. , *Deep Residual Learning for Image Recognition. Proceedings of the IEEE Conference on Computer Vision and Pattern Recognition*, 2016.

[53] Prabha A. J. , Venkatesan C. , Fathimal M. S. , Nithiyanantham K. K. , and Kirubha S. P. A. , RD-OCT Net: Hybrid Learning System for Automated Diagnosis of Macular Diseases From OCT Retinal Images, Biomedical Physics & Engineering Express. (2024) 10, no. 2, 10.1088/2057-1976/ad27ea. 38335542

[54] Gencer G. and Gencer K. , Advanced Retinal Disease Detection From OCT Images Using a Hybrid Squeeze and Excitation Enhanced Model, PLoS One. (2025) 20, no. 2, 10.1371/journal.pone.0318657. PMC11805419 39919140

[55] Mokhashi N. , Grachevskaya J. , Cheng L. et al., A Comparison of Artificial Intelligence and Human Diabetic Retinal Image Interpretation in an Urban Health System, Journal of Diabetes Science and Technology. (2022) 16, no. 4, 1003–1007, 10.1177/1932296821999370. 33719599 PMC9264425

[56] Lim J. I. , Regillo C. D. , Sadda S. R. et al., Artificial Intelligence Detection of Diabetic Retinopathy: Subgroup Comparison of the EyeArt System With Ophthalmologists’ Dilated Examinations, Ophthalmology Science. (2023) 3, no. 1, 10.1016/j.xops.2022.100228. PMC9636573 36345378

[57] Łajczak P. and Łajczak A. , Sharper Than Human Eyes? A Systematic Review and Meta-Analysis of Machine Learning for Retinal Detachment Detection, European Journal of Ophthalmology. (2026) 10.1177/11206721261419673. 41666118

[58] Keenan T. D. L. , Clemons T. E. , Domalpally A. et al., Retinal Specialist Versus Artificial Intelligence Detection of Retinal Fluid From OCT: Age-Related Eye Disease Study 2: 10-Year Follow-on Study, Ophthalmology. (2021) 128, no. 1, 100–109. 32598950 10.1016/j.ophtha.2020.06.038 PMC8371700

[59] Zatreanu L. , Freund K. B. , Leong B. C. S. et al., Serous Macular Detachment in Best Disease: A Masquerade Syndrome, Retina. (2020) 40, no. 8, 1456–1470, 10.1097/iae.0000000000002659. 31613838

[60] Rezaei-Dastjerdehei M. R. , Mijani A. , and Fatemizadeh E. , Addressing Imbalance in Multi-Label Classification Using Weighted Cross Entropy Loss Function, *2020 27th National and 5th International Iranian Conference on Biomedical Engineering (ICBME)*, November 2020.

[61] Ren J. , Wang Y. , Mao M. , and Cheung Y.-M. , Equalization Ensemble for Large Scale Highly Imbalanced Data Classification, Knowledge-Based Systems. (2022) 242, 10.1016/j.knosys.2022.108295.

[62] Selvaraju R. R. , Das A. , Vedantam R. , Cogswell M. , Parikh D. , and Batra D. , Grad-CAM: Why Did You Say That?, 2016, arXiv preprint arXiv:161107450.

