# Supplementary material for: Classification of Inherited Retinal Diseases Using Artificial Intelligence Models for Fundus Autofluorescence and Ultrawide Retinal Images
Source: J Ophthalmol. 2026 Jul 31;2026:8810684. doi: 10.1155/joph/8810684 (PMC13428240; doi:10.1155/joph/8810684)
Supplement: Supplementary file 1 — Supporting Information Table S1. Ablation study of RETFound components: effect of imaging modality, augmentation, synthetic data and MixUp on classification performance. Each cell reports the metric value obtained by the corresponding RETFound configuration; the signed change (Δ) relative to the full baseline (top row, shaded) is shown in parentheses beneath in green when the ablation improves and red when it degrades performance. The first block varies the input imaging modality (FAF‐only and RG‐only); the second block (shaded) removes one training component at a time (augmentation, synthetic data or MixUp). The best value in each metric column is shown in bold. FAF = fundus autofluorescence; RG = red–green channels; Aug = data augmentation; Syn = synthetic data; κ = Cohen’s kappa; MCC = Matthews correlation coefficient; AUC Macro is the macro‐averaged one‐vs.‐rest ROC AUC. [file JOPH-2026-8810684-s001.docx]

**Supplementary Material**

**Classification of inherited retinal diseases using artificial intelligence models for fundus autofluorescence and ultrawide retinal images**

Han Trinh, Ibrahim Muhammed, Jason Charng, Zahra Tajbakhsh, Fred K. Chen, Ajmal Mian, Khyber Alam

**Ablation study**

The full model configuration (FAF + RG fusion with all augmentations and synthetic data) achieved the highest AUC (0.945), and accuracy of 0.815. Removing augmentation reduced accuracy to 0.797 and F1 to 0.699. Removing synthetic data generation decreased F1 from 0.730 to 0.708. Modality ablation revealed that multimodal fusion (FAF + RG) outperformed both single-modality configurations: RG-only achieved accuracy of 0.770 and AUC of 0.909, while FAF-only achieved accuracy of 0.765 but a substantially lower AUC of 0.727. Removing MixUp resulted in the highest accuracy (0.835) and F1 (0.779) among all ablation variants.

Table S1. Ablation study of RETFound components: effect of imaging modality, augmentation, synthetic data, and MixUp on classification performance. Each cell reports the metric value obtained by the corresponding RETFound configuration; the signed change (Δ) relative to the Full baseline (top row, shaded) is shown in parentheses beneath in green when the ablation improves and red when it degrades performance. The first block varies the input imaging modality (FAF-only and RG-only); the second block (shaded) removes one training component at a time (augmentation, synthetic data, or MixUp). The best value in each metric column is shown in bold. FAF = fundus autofluorescence; RG = red–green channels; Aug = data augmentation; Syn = synthetic data; κ = Cohen’s kappa; MCC = Matthews correlation coefficient; AUC Macro is the macro-averaged one-vs-rest ROC AUC.

| **Configuration** | **Accuracy** | **F1 Macro** | **F1 Weighted** | **AUC Macro** | **Cohen’s κ** | **MCC** |
| --- | --- | --- | --- | --- | --- | --- |
| **Full (FAF + RG + Aug + Syn)** | 0.815 | 0.730 | 0.818 | 0.945 | 0.740 | 0.745 |
| FAF Only | 0.765  (−0.049) | 0.684  (−0.046) | 0.765  (−0.053) | 0.727  (−0.218) | 0.688  (−0.052) | 0.701  (−0.044) |
| RG Only | 0.770  (−0.045) | 0.665  (−0.065) | 0.768  (−0.051) | 0.909  (−0.037) | 0.670  (−0.070) | 0.679  (−0.066) |
| (−) Augmentation | 0.797  (−0.018) | 0.699  (−0.030) | 0.800  (−0.018) | 0.943  (−0.003) | 0.718  (−0.022) | 0.724  (−0.021) |
| (−) Synthetic data | 0.810  (−0.005) | 0.708  (−0.022) | 0.809  (−0.009) | **0.955**  (+0.009) | 0.734  (−0.006) | 0.741  (−0.004) |
| (−) MixUp | **0.835**  (+0.021) | **0.779**  (+0.049) | **0.840**  (+0.022) | 0.954  (+0.009) | **0.770**  (+0.031) | **0.776**  (+0.032) |
